# Neoantigens in cancer immunotherapy: quantity vs. quality

**DOI:** 10.1002/1878-0261.13483

**Published:** 2023-07-10

**Authors:** Yochai Wolf, Yardena Sameuls

**Affiliations:** ^1^ Ella Lemelbaum Institute for Immuno‐oncology Sheba Medical Center Ramat Gan Israel; ^2^ Department of Pathology, Faculty of Medicine Tel Aviv University Tel Aviv Israel; ^3^ Department of Molecular Cell Biology Weizmann Institute of Science Rehovot Israel

**Keywords:** cancer immunotherapy, cancer microbiome, neoantigens, post‐translational modifications, recurrent mutations, tumor heterogeneity

## Abstract

Traditional immunotherapies provide clinical benefits to only a few patients with solid tumors, highlighting the urgent need for more effective approaches. Traditional immunotherapies rely on the presentation of cancer antigens, with neoantigens being highly important in this context as they are specific to malignant tissue but not healthy tissue. The quantity of neoantigens is often associated with clinical benefit, but it cannot fully explain or predict patient response. In this Viewpoint, we highlight several qualitative aspects that should be considered in neoantigen‐based therapy. We emphasize the distinction between private and recurrent neoantigens, discuss the importance of neoantigen clonality, and describe new subtypes of neopeptides that further diversify the potential of neoantigens in immunotherapy.

## Graphical abstract

Immunotherapy largely relies on the immune system's recognition of neopeptides. In particular, cancer vaccines targeting neopeptides have shown promising clinical results. Yet, to optimize their efficacy and broaden their application, we need to decipher the nature of the cancer peptide landscape and delineate the neopeptide properties required to induce a potent immune response.

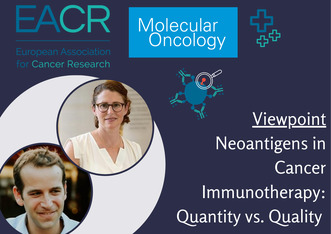

**Abbreviations**
ACT: adoptive cell transfer
HLA: human leukocyte antigens
ICB: immune checkpoint blockade
TCR: T‐cell receptor
TMB: tumor mutational burden

Cancer immunotherapy for solid tumors, whether immune checkpoint blockade (ICB) or adoptive cell transfer (ACT) of tumor‐infiltrating lymphocyte products, has brought new hope to oncology due to its remarkable ability to induce long‐term tumor regression in metastatic cancer. However, most tumors do not respond to immunotherapy, and the determinants of treatment efficacy remain largely unknown, with the search for routes to higher effectiveness being highly sought after [1]. At the core of the antitumor immune response lies the recognition of human leukocyte antigen (HLA)‐bound tumor antigens by T‐cell receptors (TCRs). A specific class of tumor antigens derived from somatic mutations, known as neoantigens, has an exceptionally high potential for future cancer treatment. Neoantigens are cell‐surface peptide/HLA complexes where the peptide component, called the neopeptide, is the altered degradation product of a nonsynonymous mutated protein. Restricted to expression in diseased tissue and unaffected by immune tolerance, neoantigens may elicit specific antitumor reactivity upon TCR engagement, making them ideal therapeutic targets. It is essential to distinguish between ‘private’ neoantigens, usually passenger mutations restricted to individual patients, and ‘public’ or ‘hotspot’ neoantigens, which stem from driver mutations in oncogenes and are prevalent in many cancer patients across various cancer types.

Personalized neoantigen‐based cancer vaccines [2, 3, 4] and TCRs targeting ‘hotspot’ mutations are already being tested in clinical trials with promising results [5, 6]. However, a key question in the neoantigen field is how to select the target neoantigen(s). The prevailing paradigm is that high tumor mutational burden (TMB), indicating a high quantity of potential neoantigens, is associated with clinical benefit from immunotherapy, despite several shortcomings with this notion, such as the benefit of immunotherapy in low TMB tumors (discussed in Ref. [7]). However, the observation that a few potent neoantigens, or even a single one, are sufficient to induce significant tumor regressions through ACT demonstrates the principle that the quality, rather than the quantity, of neoantigens will determine the therapeutic outcome for an individual.

A decade ago, the main focus of the neoantigen field was to query for passenger mutation‐derived neoantigens [8]. Most neoantigens derive from private mutations and, thus, cannot be generalized beyond the individual patient. However, most identified neoantigens stem from passenger mutations, making them unuseful beyond the individual patient. Neoantigen‐targeting therapies are, therefore ultra‐personalized to date, limiting their widespread applicability. In contrast, ACT targeting of a single potent, recurrent neoantigen may provide significant clinical benefit even in cancer types that notoriously do not respond to ICB [5, 6]. Targeting such neoantigens not only benefits many more cancer patients but also offers relevant targets across different cancer types (for instance, the KRAS^G12D^ mutation, shared between colorectal cancer and pancreatic cancer, or the NRAS^Q61K^ mutation, which is shared between melanoma and multiple myeloma) potentially paving the way for ‘off‐the‐shelf’ cellular treatments, vaccines, and patient screening strategies.

Another critical aspect is the clonality of neoantigens. Genetic intratumor heterogeneity, manifested by the distribution of clonal versus subclonal mutations and neoantigens, proved to be a significant determinant of immunotherapy response and overall prognosis in recent years [9]. Additionally, clonal TMB is a stronger predictor of ICB response than total TMB. In contrast, high subclonal TMB, in heterogeneous tumors, makes for an ineffective antitumor response [10]. Recurrent neoantigens, based on driver mutations which are by definition clonal genetic events, are thus not only highly prevalent but also highly clonal. However, since these mutations are so fundamental to cancer progression and appear in many of the cancer cells, there is a strong evolutionary pressure to suppress neoantigen presentation as a mechanism of immune escape, making the detection of efficient TCRs against them challenging.

Finally, the neoantigen field keeps expanding, and new types of noncanonical neoantigens which do not stem from nonsynonymous mutations are being discovered. These include neoantigens derived from insertions/deletions (indels), which could yield completely foreign neopeptides that are vastly different than the wild‐type form, and thus might be highly immunogenic. Nevertheless, their clonality could vary and their cross‐patient and cross‐cancer potential is unknown [11]. Neopeptides not encoded in the genomes but derived from translation aberrations [12] and post‐translational modification, through their ability to elicit specific T‐cell response is yet to be elucidated [13]. Finally, following the recent discovery of tumor‐residing microbiome, microbial peptides were also shown for their immunoreactivity [14]. These observations open new horizons and opportunities in cancer immunology and immunotherapy.

In conclusion, unlike the previous paradigm that emphasized the sheer quantity of neoantigens and drew useful but imperfect associations between quantity and clinical benefit, it is paramount that the quality of the presented antigens is considered clinically as well. Moreover, the discovery of new types of neoantigens in the ever‐evolving field of immunotherapy may prove to be valuable in identifying novel candidates for new and improved therapeutics, potentially breaking current barriers in cancer therapy.

## Conflict of interest

Y.W. receives a research grant from Pfizer, which is not related to the subject of this review.
